# Recurrent Volvulus during Pregnancy: Case Report and Review of the Literature

**DOI:** 10.1155/2018/4510754

**Published:** 2018-03-01

**Authors:** Layan Alrahmani, Jaclyn Rivington, Carl H. Rose

**Affiliations:** ^1^Mayo Clinic, Division of Maternal-Fetal Medicine, Department of Obstetrics and Gynecology, 200 First St. SW, Rochester, MN 55905, USA; ^2^Metrohealth Medical Center, Department of Internal Medicine, 2500 Metrohealth Dr., Cleveland, OH 44109, USA

## Abstract

**Introduction:**

Sigmoid colon volvulus (SV) represents the most common etiology of antepartum gastrointestinal obstruction, with repetitive antepartum episodes rarely reported.

**Case Presentation:**

A 25-year-old multiparous patient with history of SV at 26 weeks in her previous pregnancy presented with recurrent episodes of SV at 32 0/7, 32 4/7, 37 0/7, and 38 1/7 weeks successfully managed with colonoscopic decompression. Labor was successfully induced at 38 4/7 weeks, and she experienced two further episodes on postpartum days #1 and #32 also treated with colonoscopic decompression, followed by laparoscopic resection.

**Conclusion:**

Successful treatment of antepartum SV with colonoscopic decompression does not preclude recurrence later in gestation and in future pregnancies.

## 1. Background

Volvulus of the sigmoid colon represents the most common etiology of antepartum bowel obstruction [1, 2]. Circumferential torsion of an intestinal segment around its mesenteric origin produces symptoms of gastrointestinal colic, and if untreated may progress to bowel ischemia and perforation [1, 2]. The increase in uterine size accompanying advancing gestational age with subsequent cephalad displacement of pelvic segment of the colon has been proposed as a risk factor; most reported cases are isolated solitary occurrences, with recurrent episodes only rarely described. In this report, we present a case with recurrent sigmoid volvulus (SV) in two successive pregnancies treated with temporizing endoscopic decompression and elective postpartum sigmoid resection.

## 2. Case Presentation

A 25-year-old gravida 3, para 2002 at 32 0/7 weeks' gestation was referred with a 24-hour history of intermittent severe abdominal pain and obstipation. Her past medical history was significant for hypothyroidism, a microcytic anemia, and sigmoid volvulus in her previous pregnancy two years prior requiring endoscopic reduction at 26 weeks' gestation. Physical examination revealed suprapubic and right lower quadrant tenderness, with normal bowel sounds on auscultation; peritoneal signs of rebound and/or guarding were absent. Obstetric ultrasound demonstrated a nonanomalous singleton fetus with biometry consistent with gestational age, no evidence of placental abruption, and normal cervix length. Due to gravid status, abdominal radiography was inconclusive but suggestive of colonic distention. Laboratory evaluation was remarkable for normal leukocyte count and lactate, amylase, and hepatic transaminase levels. Two tap water enemas were efficacious in prompting adequate stool output with alleviation of clinical symptoms, and she was discharged.

**Figure 1:**
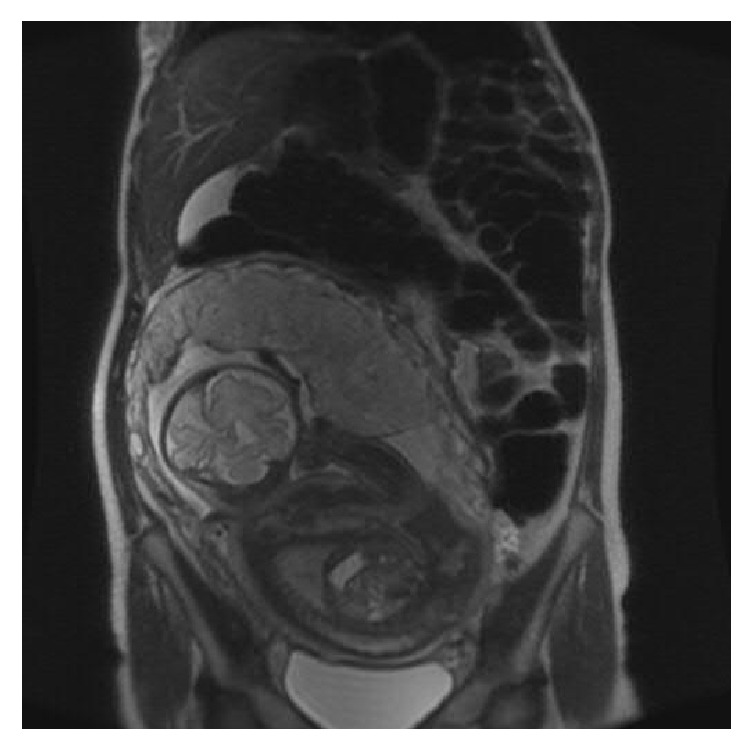
Magnetic resonance imaging without contrast performed at 32 weeks' gestation. There is marked distention of the sigmoid colon beginning at the level of the sigmoid along the left lateral aspect of the uterus where there appears to be a small, early (90 degree) twist of the sigmoid colon. The fetus may also be seen in this image.

**Figure 2:**
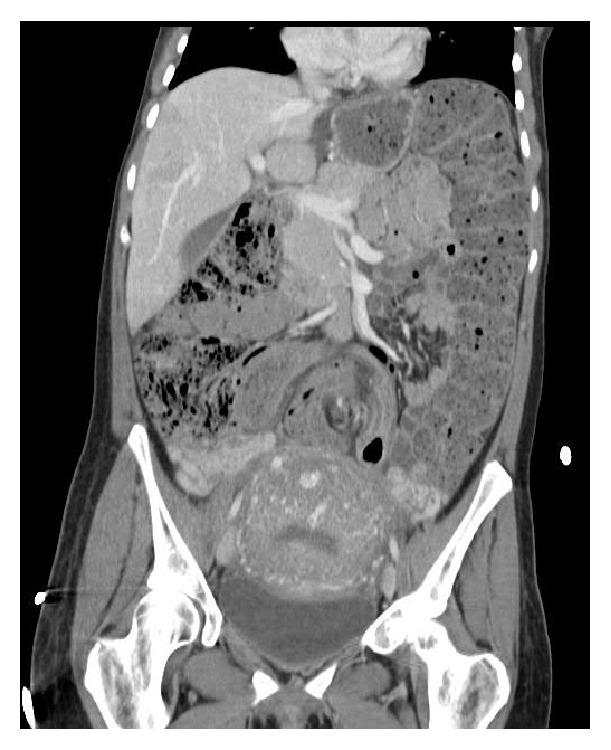
Computer tomography imaging with intravascular contrast performed on postpartum day 1. There is twisting of the mesentery and focal mesenteric edema in the mid lower abdomen. The findings are consistent with sigmoid volvulus.

Subsequently, she returned at 32 4/7 weeks with similar complaints; the gastroenterology service was consulted, and magnetic resonance imaging confirmed a 90-degree twist of the colon 30 cm from the anal verge (Figure 1). Colonoscopic decompression followed by rectal tube placement for 24 hours was successful, and she was discharged home. Unfortunately, symptoms recurred at 37 0/7 weeks, and she underwent a second colonoscopy with decompression, with rectal tube left in place. At 38 1/7 weeks, she underwent a third colonoscopic decompression procedure, and the decision was made to proceed with delivery. Labor was induced, culminating in a spontaneous vaginal delivery of a healthy female infant. Unfortunately she again experienced worsening abdominal pain on the first day postpartum, with computer tomography imaging confirming sigmoid distention with recurrence of volvulus (Figure 2). A fourth colonoscopic decompression was performed, and she was discharged home on the second day postpartum. Symptoms again recurred on postpartum day 32 and were managed by a fifth colonoscopic decompression followed by uncomplicated laparoscopic sigmoidectomy on postpartum day 34. Histopathologic evaluation of the excised specimen was benign.

## 3. Discussion and Conclusion

A literature search of the PubMed, Google Scholar, Scopus, and the Cochrane Library databases from January, 01, 1900, to January, 22, 2017, was performed using search terms “recurrent”, “sigmoid volvulus”, and “pregnancy” alone or in combination using the Boolean operator “AND”. Only patients with recurrent sigmoid volvulus in pregnancy were considered for this review. The search initially yielded 8 articles, and after careful review by two of the authors (Jaclyn Rivington, Layan Alrahmani) only 3 met the inclusion criteria of multiple episodes of sigmoid volvulus recurring in the same pregnancy; specific characteristics are summarized in Table 1.

Intestinal obstruction in pregnancy is relatively rare, with an incidence of 1 in 1500 to 1 in 66,431 deliveries. To date, only 105 cases of volvulus have been reported during pregnancy [6], with only 3 authors describing repetitive episodes. Typical risk factors include older age, high fiber diet, constipation, and an elongated redundant sigmoid colon [7, 8]; the disproportionate incidence during pregnancy is theorized to occur due to the physical size of the enlarging gravid uterus displacing the colon out of the pelvis, perhaps explanatory of the frequency of third-trimester presentation. Furthermore, elevated gestational progesterone levels cause hypomotility of the gastrointestinal tract through smooth muscle relaxation, increasing constipation and volvulus risk [3]. Age and multigravidity do not appear to constitute significant factors [9]. Although in the nonpregnant population eventual recurrence of sigmoid volvulus is considered to be approximately 50%, it has only rarely been documented in pregnancy [10].

Management of sigmoid volvulus in pregnancy is similar to the nonpregnant state. The American Society of Colon and Rectal Surgeons suggest initial rigid or flexible endoscopy to assess for intestinal viability and to effect therapeutic detorsion and decompression (Grade 1c) [11]; this is reported to be successful in 75–95% of cases [11–13]. Definitive sigmoidectomy is considered in the acute setting if there is evidence of nonviable or perforated colon, or after the resolution of the acute phase of sigmoid volvulus to prevent recurrence (Grade 1c) [11]. A decompression tube is often placed for 1–3 days after the endoscopic procedure; however the utility of this intervention has not been established [14].

Alshawi suggests that elective sigmoidectomy be considered in the second trimester of recurrent cases of SV [4], due to the potential for continued recurrence with development of bowel necrosis later in gestation. However, due to the small cohort of patients actually reported to experience antepartum recurrence of SV, the optimal course of action is difficult to conclusively extrapolate. In our case, the patient was managed with repetitive endoscopic decompressions in the third trimester, which is considered to pose a low maternal and fetal risk irrespective of gestational age [15]. The risk of elective or emergent surgical bowel resection must be weighed against the challenge of repeated endoscopic treatments; with anticipated reduction in uterine size following delivery, one could argue that the instigating factor would “resolve” postpartum, possibly eliminating the need for colon resection.

It is difficult to ascertain whether volvulus is more likely to recur in pregnancy. Since pregnancy is a predisposing factor, we can assume that since the enticing factor still exists, namely, pregnancy, the chance for recurrence is high. Time to recurrence of volvulus is variable. Prevention strategies for recurrence other than definitive surgery are unknown. Prevention of constipation with dietary modifications and stool softeners is safe but short-term efficacy is not determined.

Obstetrical management in these situations must be individualized; the authors would recommend a multidisciplinary approach involving Obstetrics, Maternal-Fetal Medicine, Anesthesiology, Gastroenterology, and Colorectal Surgery. Vaginal delivery is not contraindicated, and cesarean delivery should be reserved for routine obstetrical indications; of note, in all reported cases of recurrent SV (including the current), vaginal delivery was accomplished. Monitoring for symptoms of intrapartum recurrence, particularly if neuraxial anesthesia is elected, is important; in the event that cesarean delivery is required, depending on preoperative preparation, concurrent elective bowel resection may be considered.

Reported prognosis of SV varies widely, with maternal mortality rates of 5–12% and fetal mortality of 20–26% [1]. Almost all maternal deaths occurred when patients presented more than 48 hours following onset of symptoms. Similarly, maternal mortality associated with viable bowel is only 5% but increases to greater than 50% once bowel perforation has occurred [6], underscoring the importance of timely diagnosis and intervention. Recurrence of SV in subsequent pregnancies and/or postpartum has not previously been described; interestingly the current case occurred at a similar gestational age in two successive pregnancies, suggesting the basic underlying anatomy with the additional uterine compression as the precipitating factor. Patients experiencing SV in pregnancy successfully treated with colonoscopic decompression should be counseled on the potential for recurrence both later in gestation and in future pregnancies.

## Figures and Tables

**Table 1:** Previously published cases of recurrent sigmoid volvulus during pregnancy.

Author (year)	Patient demographic	Initial episode (weeks gestation)	Intervention	Recurrence (weeks gestation)	Intervention	Delivery	Outcome
Bajaj et al. (2015) [3]	23 yo G3P2	5	Endoscopic decompression	36 5/7	Rigid sigmoidoscopy, only partial decompression	SVD at 36 weeks	Sigmoidectomy at 6 weeks postpartum
Alshawi (2005) [4]	22 yo G2P1	20	Sigmoidoscopic detorsion and rectal tube	28, 35	Colonoscopic detorsion and rectal tube	SVD at 38 weeks	Sigmoidectomy at 2 weeks postpartum
Bandler et al. (1964) [5]	28 yo G1P0	16	Laparotomy with derotation and pinning of sigmoid	37 1/7	Derotation during CD	CD at 37 weeks	Subtotal colectomy at 4 weeks postpartum
Our case	25 yo G3P2	32	Sigmoidoscopic detorsion and rectal tube	32 5/7, 37, 38 1/7	Endoscopic decompression and rectal tube	SVD at 38 weeks	Sigmoid colectomy at 4 weeks postpartum

CD, cesarean delivery; SVD, spontaneous vaginal delivery.

## Data Availability

Data sharing is not applicable to this article as no datasets were generated or analysed during the current study.

## Abbreviations

SV: Sigmoid volvulus.

## References

[1] Aftab Z., Toro A., Abdelaal A. (2014). Endoscopic reduction of a volvulus of the sigmoid colon in pregnancy: case report and a comprehensive review of the literature. *World Journal of Emergency Surgery (WJES)*.

[2] Khan M. R., ur Rehman S. (2012). Sigmoid volvulus in pregnancy and puerperium: a surgical and obstetric catastrophe. Report of a case and review of the world literature. *World Journal of Emergency Surgery*.

[3] Bajaj M., Gillespie C., Dale J. (2015). Recurrent sigmoid volvulus in pregnancy. *ANZ Journal of Surgery*.

[4] Alshawi J. S. (2005). Recurrent sigmoid volvulus in pregnancy: Report of a case and review of the literature. *Diseases of the Colon & Rectum*.

[5] Bandler M., Freidman S., Roberts M. (1964). Recurrent volvulus of the sigmoid colon during pregnancy complicated by toxemia of pregnancy. *The American Journal of Gastroenterology*.

[6] Al Maksoud A. M., Barsoum A. K., Moneer M. M. (2015). Surgery case reports signet ring cell carcinoma of the ampulla of vater?: report of a case and a review of the literature. *International Journal of Surgery Case Reports*.

[7] Madiba T. E., Aldous C., Haffajee M. R. (2015). The morphology of the foetal sigmoid colon in the African population: A possible predisposition to sigmoid volvulus. *Colorectal Disease*.

[8] Michael S. A., Rabi S. (2015). Morphology of sigmoid colon in south indian population: a cadaveric study. *Journal of Clinical and Diagnostic Research*.

[9] Harer W. B. Jr., Harer W. B. Sr. (1958). Volvulus complicating pregnancy and puerperium: report of three cases and review of literature. *Obstetrics & Gynecology*.

[10] Mangiante E. C., Croce M. A., Fabian T. C., Moore O. F., Britt L. G. (1989). Sigmoid volvulus. A four-decade experience. *The American Surgeon*.

[11] Vogel J. D., Feingold D. L., Stewart D. B. (2016). Clinical Practice Guidelines for Colon Volvulus and Acute Colonic Pseudo-Obstruction. *Diseases of the Colon and Rectum*.

[12] Atamanalp S. S. (2013). Treatment of sigmoid volvulus: A single-center experience of 952 patients over 46.5 years. *Techniques in Coloproctology*.

[13] Anderson J. R., Lee D. (1981). The management of acute sigmoid volvulus. *British Journal of Surgery*.

[14] Harrison M. E., Anderson M. A., Appalaneni V. (2010). The role of endoscopy in the management of patients with known and suspected colonic obstruction and pseudo-obstruction. *Gastrointestinal Endoscopy*.

[15] De Lima A., Galjart B., Wisse P. H. A. (2015). Does lower gastrointestinal endoscopy during pregnancy pose a risk for mother and child? - a systematic review. *BMC Gastroenterology*.

